# iStent with Phacoemulsification versus Phacoemulsification Alone for Patients with Glaucoma and Cataract: A Meta-Analysis

**DOI:** 10.1371/journal.pone.0131770

**Published:** 2015-07-06

**Authors:** Monali S. Malvankar-Mehta, Yiannis Iordanous, Yufeng Nancy Chen, Wan Wendy Wang, Sangita Shantilal Patel, John Costella, Cindy M. L. Hutnik

**Affiliations:** 1 Department of Ophthalmology, Schulich School of Medicine and Dentistry, University of Western Ontario, London, ON, Canada; 2 Department of Epidemiology and Biostatistics, Schulich School of Medicine and Dentistry, University of Western Ontario, London, ON, Canada; 3 Schulich School of Medicine and Dentistry, University of Western Ontario, London, ON, Canada; 4 Faculty of Health Sciences, University of Western Ontario, London, ON, Canada; 5 Allyn & Betty Taylor Library, Natural Sciences Centre, University of Western Ontario, London, ON, Canada; 6 Department of Pathology, Schulich School of Medicine and Dentistry, University of Western Ontario, London, ON, Canada; Casey Eye Institute, UNITED STATES

## Abstract

**Background:**

Minimally invasive glaucoma surgeries (MIGS) have attracted significant attention, as they have been reported to lower intra-ocular pressure (IOP) and have an excellent safety profile. The iStent is an example of a minimally invasive glaucoma device that has received particular attention due to its early and wide spread utilization. There is a growing body of evidence supporting its use at the time of phacoemulsification to help lower IOP. However, it is still not clear how much of the IOP lowering effect can be attributed to the iStent, the crystalline lens extraction or both when inserted concurrently at the time of phacoemulsification. This has been an important issue in understanding its potential role in the glaucoma management paradigm.

**Purpose:**

To conduct a systematic review and meta-analysis comparing the IOP lowering effect of iStent insertion at the time of phacoemulsification versus phacoemulsification alone for patients with glaucoma and cataracts.

**Methods:**

A systematic review was conducted utilizing various databases. Studies examining the IOP lowering effect of iStent insertion in combination with phacoemulsification, as well as studies examining the IOP lowering effect of phacoemulsification alone were included. Thirty-seven studies, reporting on 2495 patients, met the inclusion criteria. The percentage reduction in IOP (IOPR%) and mean reduction in topical glaucoma medications after surgery were determined. The standardized mean difference (SMD) was computed as a measure of the treatment effect for continuous outcomes taking into account heterogeneity. Fixed-effect and random-effect models were applied.

**Results:**

A 4% IOP reduction (IOPR%) from baseline occurred following phacoemulsification as a solo procedure compared to 9% following an iStent implant with phacoemulsification, and 27% following 2 iStents implants with phacoemulsification. Compared with cataract extraction alone, iStent with phacoemulsification resulted in significant reduction in the post-operative IOP (IOPR%) (SMD = -0.46, 95% CI: [-0.87, -0.06]). A weighted mean reduction in the number of glaucoma medications per patient was 1.01 following phacoemulsification alone compared to 1.33 after one iStent implant with phacoemulsification, and 1.1 after 2 iStent implants with phacoemulsification. Compared to cataract extraction alone, iStent with cataract extraction showed a significant decrease in the number of glaucoma medications (SMD = -0.65, 95% CI: [-1.18, -0.12]). Funnel plots suggested the absence of publication bias.

**Conclusion:**

Both iStent implantation with concurrent phacoemulsification and phacoemulsification alone result in a significant decrease in IOP and topical glaucoma medications. In terms of both reductions, iStent with phacoemulsification significantly outperforms phacoemulsification alone.

## Introduction

Open angle glaucoma (OAG) is the leading cause of irreversible vision loss[1,2] affecting 60.5 million people globally. The prevalence of OAG is projected to rise to nearly 80 million people by 2020.[3,4] OAG is an enormous public health burden, with the direct annual cost of treatment estimated at 2.86 billion (USD) in the U.S. [5] and 300 million (CAD) in Canada [6] including productivity loss. These costs are cumulative and can have a huge financial impact for patients, care givers, and for society at large.[7] Additionally, the social consequences of severe OAG includes difficulties in performing daily activities, increased risk of falls[8], injuries, depression [9], hip fractures [10], and increased risk of death [1]. Compared to people with normal vision, people with low vision are prematurely admitted to nursing homes.[11]

At present, the only known treatment to lower the risk of visual loss secondary to glaucoma is lowering of the intraocular pressure (IOP). In the traditional treatment paradigm, topical medications are the first-line of therapy for OAG. Despite proven efficacy and a good safety profile, there are a number of issues with medications. Compliance is a major concern with any chronic medication use.[12–14] It has been estimated that only 67% of patients are compliant with topical glaucoma medications [15] and that rate decreases when multiple medications are utilized.[16] Side-effects such as ocular and peri-ocular erythema, transient blurred vision, itching, local irritation, and ocular surface toxicity [17–19], eye discomfort, photosensitivity, increased tears, burning and stinging, eyelash changes, hyperpigmentation of the eyelid and irides have been associated with poor compliance to topical medications.[20] Additionally, medications can be difficult to administer.[21,22]

Micro-invasive glaucoma surgery (MIGS) and devices have been gaining increasing attention in recent years. One such device is the trabecular microbypass stent, or iStent (Glaukos Corporation, Laguna Hills, CA). It is the smallest implantable medical device which is inserted *ab interno* into Schlemm’s canal [23], causing a lowering of IOP.[24,25] Part of the appeal of the iStent is that it circumvents issues related to patient compliance, and has a favorable safety profile. In addition, a cost-analysis suggests that the iStent may also be more cost effective than topical medications.[7] There are emerging data supporting the use of iStent as an adjunctive therapy at the time of phacoemulsification for patients who are on multiple glaucoma medications.[24,26–30]

Most of the studies examining the effect of iStent on IOP have been small or have lacked an adequate control arm. The lack of a control arm is a concern, as phacoemulsification alone has been found to lower IOP in both short and long term studies.[31–33] The primary objective of the current systematic review and meta-analysis is to compare the IOP lowering effect of iStent insertion at the time of phacoemulsification versus phacoemulsification alone for patients with glaucoma and cataract. A secondary aim is to examine visual acuity outcomes and topical glaucoma medication use for both groups.

## Methods

### Literature Retrieval

We adhered to the Preferred Items for Systematic Reviews and Meta-Analyses (PRISMA) guidelines (S1 File).[34] Computer databases including MEDLINE (OVID and Pubmed), EMBASE (OVID), BIOSIS Previews (Thomson-Reuters), CINAHL (EBSCO), Health Economic Evaluations Database (HEED), ISI Web of Science (Thomson-Reuters) and the Cochrane Library (Wiley) were searched from the year 2000 to June 2014. The reference lists of all the included articles were hand searched to find potentially relevant studies. Grey literature was identified by searching the conference abstracts of various meetings including the Canadian Ophthalmology Society meeting (COS), American Academy of Ophthalmology annual meeting (AAO), European Society of Ophthalmology (SOE), American Glaucoma Society (AGS), and the Association for Research in Vision and Ophthalmology annual meeting (ARVO). The Proquest Dissertations and Theses databases and the Canadian Health Research Collection (Ebrary) were also searched for relevant content. Google and other internet search engines were used to search for additional web-based materials and information. OVID AutoAlerts were set up to send monthly updates with any new literature.

Database specific subject headings and key words for “phacoemulsification”, “iStent”, “open angle glaucoma”, “ocular hypertension” were employed in the search strategy. The searches were modified to accommodate the unique terminology and syntax of each database. Detailed search strategy for EMBASE has been provided in the supplementary material (S2 File). Searches were limited to human subjects and studies published between January 2000 and June 2014. Year 2000 was selected to be consistent with the way phacoemulsification surgeries are performed.

### Inclusion and Exclusion Criteria

A minimum post-op period of two months was required for inclusion in order to limit the confounding effects of short-term fluctuations in IOP that can occur in the immediate post-operative period due factors such as retained viscoadaptive devices. Only studies published in the English language were included. Research studies such as journal articles, systematic reviews, meta-analysis, cost analysis, cost-utility analysis, cost-effectiveness analysis, multicenter studies, randomized controlled trials, quasi- randomized controlled trials, non-randomized studies including cohort studies (retrospective, prospective), clinical trials, and comparatives studies, were included for analysis. Editorial opinions, case reports, or reviews were excluded from this analysis. A minimum sample size of 20 patients was required for inclusion. Additionally, the study population included adults above the age of 18 with OAG or ocular hypertension. Studies examining subjects with other types of glaucoma were excluded.

All the records identified through database searching and grey literature search were imported to EPPI-Reviewer 4 gateway (by EPPI-Centre, Social Science Research Unit, the Institute of Education, University of London, UK). Duplicate records were removed and the included records were screened. Screening was done at three levels. Titles of the included records were screened at Level 1; abstracts of the records included in Level 1 were screened in Level 2; and in Level 3, full texts of the included articles were screened. Detailed screening questions are provided in the supplementary material (S3 File). Two reviewers independently screened the records at each level. Cohen’s kappa (κ) coefficient was computed to assess the agreement of inclusion between the two reviewers after each level of screening. Articles were included for next level of screening if two reviewers agreed. In the case of disagreements, a third reviewer intervened to provide a decision.

### Data Extraction

One reviewer extracted the data from eligible articles and a second reviewer verified the data extracted. Study data included study objective, design, location, inclusion and exclusion criteria, data collection technique, data collection period, follow-up, intervention performed such as phacoemulsification only or iStent with phacoemulsification or both, and number of iStents inserted. Additionally, data on total patients enrolled in the study, total patients completing the study, refusal to consent, and patient demographic characteristics such as mean age, standard deviation (SD), gender, race and ethnicity (%) were collected from each included article. Baseline and post-operative characteristics such as best visual acuity corrected (BCVA), uncorrected visual acuity, IOP, number of glaucoma medications, reported complications and their rates, percentage of patients off-medications, patients with 20/40 or better vision, patients with IOP ≤ 18 mmHg post-operatively, rate of iStent malposition, iStent occlusion, and allergic reaction to glaucoma medications were also obtained.

The available data on range, *p*-value, and confidence interval were utilized and converted to the common effect measure, SD. Further, means and measures of dispersion were approximated from figures if required. Two reviewers assessed the methodological quality of each included study using the Downs and Black checklist.[35]

### Statistical Analysis

Meta-analysis was performed using STATA v. 13.0 (STATA Corporation, College Station, TX). The abstracted mean and standard error of the IOP at baseline and end-time point were used to compute the mean IOP reduction (*IOPR*), percentage of IOP reduction (*IOPR%*), within group standard error (*SE*_*IOPR*_), and standard error of percentage of IOP reduction (*SE*_*IOPR%*_) using the equations below.[36]

$$\begin{array}{l} IOPR=IOP_{baseline}–IOP_{endpoint}\\ IOPR\%=IOPR/IOP_{baseline}\\ SE_{IOPR}={\left[{\left(SE_{baseline}\right)}^{2}–{\left(SE_{endpoint}\right)}^{2}\right]}^{1/2} \end{array}$$

$$SE_{IOPR\%}=SE_{IOPR}/IOP_{baseline}$$

Standard deviation percentage of IOP reduction (*SD*_*IOPR%*_) was then calculated by the formula *SD*_*IOPR%*_ = *SE*_*IOPR%*_ x *n* ^1/2^.

For continuous scale outcomes including mean values, the standardized mean difference (SMD) was calculated as the treatment effect or effect size. This parameter represents the mean difference standardized for variances across all studies. To compute SMD for each study, the mean pre- and post-operative values for each outcome measure was divided by the SD for that same outcome measure. Weights were assigned to each SMD according to the inverse of its variance and then average was computed. The SMD in each study was pooled with a fixed- or random-effect model based on heterogeneity. To test heterogeneity, a Z-value was computed to test the null hypothesis which was a treatment effect of zero.

Additionally, heterogeneity was determined using the I^2^ value[37], which indicated the extent of variation across studies due to heterogeneity rather than chance. Heterogeneity between studies was examined using a chi-squared test, where the chi-squared test assessed whether the observed between-studies differences were due to chance only.[38] Low p-value and a large chi-squared statistic relative to its degree of freedom provided evidence of heterogeneity. Funnel plots were generated to check publication bias.

SMD of the mean post-operative number of topical glaucoma medications was computed to evaluate the impact of “iStent with phacoemulsification” versus “phacoemulsification only” on number of topical glaucoma medications. For the sub-group analysis, SMD of the mean pre-operative and post-operative number of medications was computed for phacoemulsification only, one iStent with phacoemulsification, 2 iStents with phacoemulsification, and 3 iStents with phacoemulsification. Additionally, sub-group analysis was conducted by follow-up period for “phacoemulsification only” group and for “iStents with phacoemulsification” group.

## Results

### Search Results

**Fig 1:**
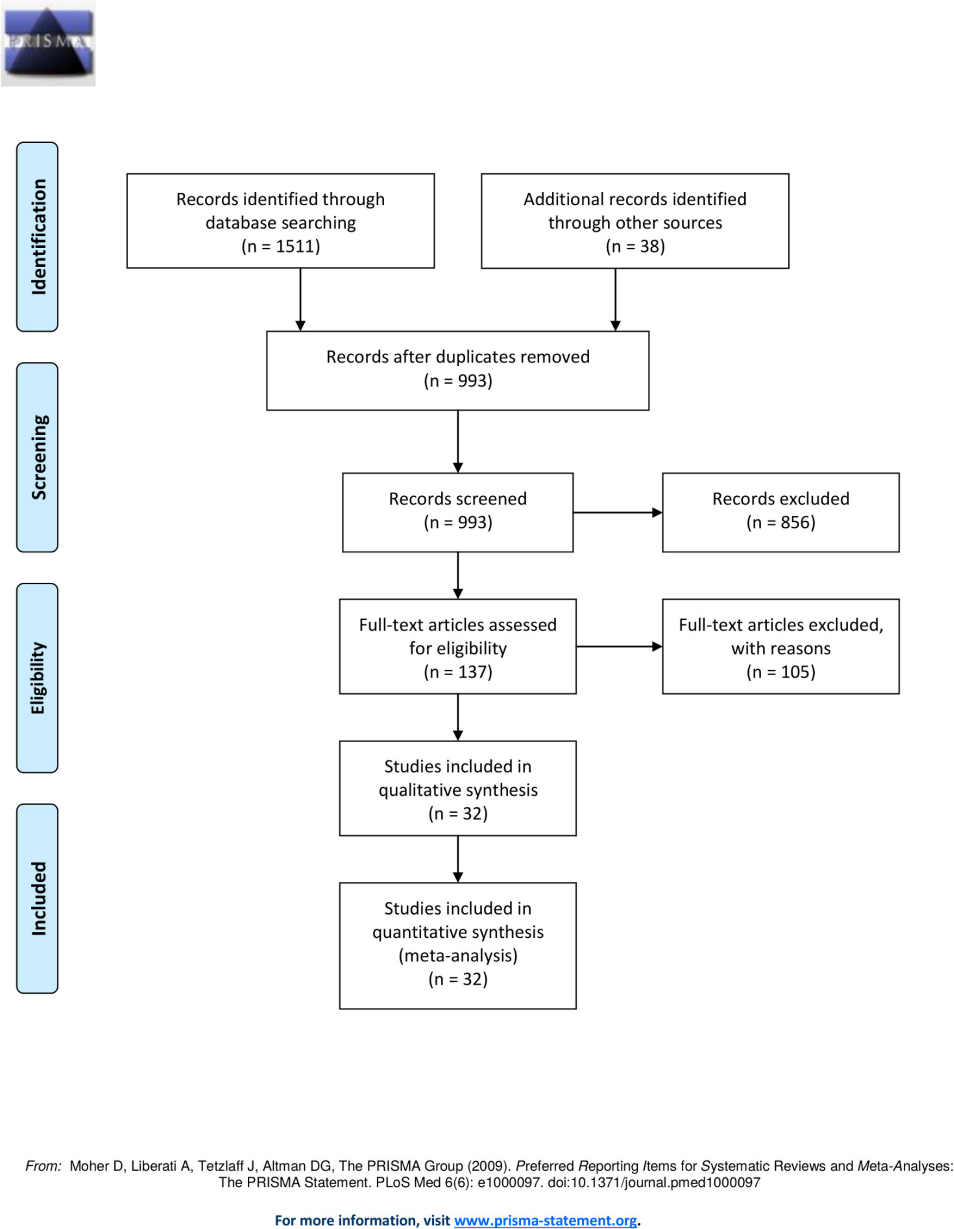
PRISMA Flow Diagram for iStent with Phacoemulsification versus Phacoemulsification alone for Patients with Glaucoma and Cataract: A Meta-Analysis.

A total of 1151 records were identified through database searching and an additional 38 records were identified through grey literature search. A total of 1189 records were imported to EPPI-Reviewer 4 and duplicate records were removed. 933 records remained for screening after removing duplicates. 147 records were retrieved after three-levels of screening. After reviewing all the full text articles, a total of 32 articles (2143 subjects) met our inclusion criteria and were utilized in our quantitative and qualitative synthesis. See Fig 1 for PRISMA flow diagram.

### Study Characteristics

Tables 1, 2, 3, 4, 5, 6, 7 and 8 summarize characteristics of studies included for meta-analysis. Table 1 lists baseline characteristics as well as pre- and post-operative IOP, IOPR%, and standard deviation of IOPR% (SD_IOPR%) of the 4 studies comparing iStent with and without concurrent phacoemulsification. Table 2 lists baseline characteristics including pre- and post-operative IOP, IOPR%, and SD_IOPR% of the 22 studies considering phacoemulsification as a solo procedure. Table 3 lists 6 studies evaluating iStent with phacoemulsification. The follow-up period varied from 2 months to 7 years. A total of 18 studies were conducted in the U.S., 4 in U.K., 3 in Italy, 1 in each country including Turkey, Spain, Germany, Finland, New Zealand, Japan, and Israel. Of the 32 studies, 21 were case series, 2 were case control, 4 were randomized control trials, and 5 were cohort studies (Table 1). Tables 4, 5, and 6 lists data on pre- and post-operative topical glaucoma medications, and mean reduction in topical glaucoma medications of the 4 studies comparing iStent with and without concurrent phacoemulsification, 22 studies considering phacoemulsification as a solo procedure, and 6 studies evaluating iStent with phacoemulsification, respectively. Table 7 lists data on pre- and post-operative best corrected visual acuity (BCVA) of the included studies. Table 8 lists data on post-operative characteristics of included studies such as complications, complication rates, percentage of patients with good vision, reduced number of medications, race and ethnicity of patients, and controlled IOP.

**Table 1:** Reported pre- and post-operative intraocular pressure in included studies comparing iStent with and without concurrent phacoemulsification.

Author (Year)	Study Design	Study Location	Intervention	N	Follow-up (months)	Mean Age	Age(SD)	Baseline IOP (Mean)	Baseline IOP (SD)	Post-operative IOP(Mean)	Post-operative IOP (SD)	IOPR%	SD_IOPR%
Fea (2010) [28]	RCT	Italy	iStent + phaco	12	15	64.9	3.1	17.9	2.6	14.8	1.2	17.32	0.1599
	RCT	Italy	Phaco only	24	15	64.5	3.4	17.3	3.0	15.7	1.1	9.25	0.1847
Fernandez-Barrientos(2010) [39]	RCT	Spain	2 iStents + phaco	17	12	75.2	7.2	24.2	1.8	17.6	2.8	27.27	0.1375
	RCT	Spain	Phaco only	16	12	-	-	23.6	1.5	19.8	2.3	16.1	0.1163
Fea(2012) [40]	RCT	Italy	iStent + phaco	10	51.3	-	-	17.8	2.7	15.9	2.3	10.67	19.93
	RCT	Italy	Phaco only	14	58.3	-	-	15.7	2.8	17	2.5	-8.28	23.91
Samuelson (2011) [26]	RCT	U.S.	Phaco only	117	12	73	0.67	18.4	3.2	17.4	1.291	5.44	18.75
	RCT	U.S.	iStent + Phaco	123	12	73	0.67	18.4	3.2	16.9	1.291	8.152	18.75

1. Based on literature [26,28,40], weighted mean reduction in IOP (IOPR%) following Phaco only was 4.7%.

2. Based on literature [26,28,40], weighted mean reduction in IOP (IOPR%) following an iStent + Phaco was 9%.

3. Based on literature [39], weighted mean reduction in IOP (IOPR%) following 2 iStents + Phaco was 27%.

**Table 2:** Reported pre- and post-operative intraocular pressure in included studies considering phacoemulsification as a solo procedure.

Author (Year)	Study Design	Study Location	N	Follow-up (months)	Mean Age	Age(SD)	Baseline IOP (Mean)	Baseline IOP (SD)	Postoperative IOP(Mean)	Postoperative IOP(SD)	IOPR%	SD_IOPR%
Altan-Yaycioglu (2009) [41]	Case Control	U. S.	26	6	70.3	7.9	21.6	7	16.5	2.9	23.61	0.351
Bowling (2009) [42]	Case series	U. K.	51	12	-	-	17.0	3.7	14.7	2.2	13.53	0.2532
Chang (2012) [43]	Case series	U. S.	29	36	75.34	7.14	15.66	3.33	14.68	3.44	6.26	0.3057
Chihara (2010) [44]	Cohort	Japan	208	6	72.3	9.3	17.6	2.7	15.9	3.9	9.66	0.2695
Damji (2006) [45]	Cohort	U. S.	29	24	73.99	10.78	18.52	3.52	16.98	3.299	8.315	0.2605
Guan (2013) [46]	Case Series	U. S.	103	6	78	6.4	15.2	3.3	13.4	2.5	11.84	27.24
Hayashi(2000) [47]	Cohort	U. S.	73	12	73.5	7.3	20.5	5.4	16.4	4.1	20	33.1
Khondkaryan (2012) [48]	Cohort	U. S.	20	24	-	-	21.4	2.9	17.5	3.7	18.22	21.97
Klink(2005) [49]	Case series	U.K.	36	12	76.2	6.8	17.59	5.26	15.58	3.15	11.43	34.86
Ismi(2013) [50]	Case Series	Turkey	21	6	69.19	9.23	14.67	3.68	12.52	2.62	14.66	30.79
Mansberger (2012) [51]	Case Series	U. S.	63	36	64.1	8.9	23.9	3.2	9.81	3.2	58.95	18.93
Mathalone (2005) [52]	Case series	Israel	24	24	80	7	17	4.6	15.1	3.2	11.18	32.96
Merkur (2001) [53]	Case Series	U. S.	23	18	78.13	6.84	17.22	3.19	15.67	2.07	9	22.08
Park (2004) [54]	Case Control	New Zealand	103	24	71.8	8.8	19.3	2.2	18.6	3	3.63	19.28
Poley (2010) [55]	Case Series	U. S.	124	12	75.53.1	-	17.8	3.28	15.4.5	1.45	13.48	20.12
Pohjalainene (2001) [56]	Case Series	Finland	38	12	78.3	6.8	18.4	3.3	15.1	2.9	17.93	23.88
Sacca (2001) [57]	Case series	Italy	15	6	63.5	9.25	19.8	3.3	15.53	2.26	21.57	20.2
Sharma (2008) [58]	Case series	U.K.	22	12	76.41	7.25	14.5	3.7	14.36	3.087	0.9	33.23
Shingleton (2006) [59]	Case Series	U.S.	55	60	77.6	7	18.4	3.4	16.6	0.831	9.78	19.02
Shingleton (2008a) [60]	Case Series	U.S.	137	12	77.6	6.6	16.4	0.3	14.1	0.5	23	16.415
Shingleton (2008b) [61]	Case Series	U.S.	240	84	78.2	7	17.4	5	16.2	6.3	6.89	46.22
Slabaugh (2014) [62]	Case Series	U. S.	157	12	74.4	10.2	16.27	3.54	14.47	3.37	11.06	30

Weighted mean reduction in IOP (IOPR%) following Phacoemulsification as a solo procedure was 31%

**Table 3:** Reported pre- and post-operative intraocular pressure in included studies considering iStent with concurrent phacoemulsification.

Author (Year)	Study Design	Study Location	Intervention	N	Follow-up (months)	Mean Age	Age(SD)	Baseline IOP (Mean)	Baseline IOP(SD)	Postoperative IOP (Mean)	Postoperative IOP (SD)	IOPR%	SD_IOPR%
Ahmed (2012) [63]	Case series	U. S.	iStent + phaco	27	6	74.2	8.9	18.6	4.5	13.3	1.7	28.49	0.2586
Arriola Villalobos (2013) [64]	Case Series	U. K.	2 iStents + Phaco	20	12	75.1	8.6	19.95	3.71	16.75	2.24	16.04	0.2172
Belovay (2012) [65]	Cohort	U. S.	2 iStents + Phaco	28	12	78.8	7	17.3	4	13.8	1.04	20.23	0.2383
			3 iStents + Phaco	25	12	75	7.3	18.6	4	14.8	1.04	20.4	0.2217
Spiegel(2008) [66]	Case Series	Germany	iStent + phaco	47	6	76.2	6.7	21.5	3.7	15.8	3	26.51	22.16
Patel (2013) [67]	Case Series	U. S.	iStent + phaco	40	12	76.8	-	21.5	5.5	16.5	4.99	23.26	34.54
Spiegel(2009) [68]	Case series	U. S.	iStent + Phaco	47	12	76.2	6.7	21.5	3.7	15.8	3.0	26.51	22.16

1. Based on the literature, weighted mean reduction in IOP (IOPR%) following an iStent + Phaco was 26%.

2. Based on the literature, weighted mean reduction in IOP (IOPR%) following 2 iStents + Phaco was 18.4%.

3. Based on the literature, weighted mean reduction in IOP (IOPR%) following 3 iStents + Phaco was 20%.

**Table 4:** Reported number of pre- and post-operative topical glaucoma medications in studies comparing iStent with and without concurrent phacoemulsification.

Author (Year)	N	Intervention	Baseline Topical Glaucoma Medications(Mean)	Baseline Topical Glaucoma Medications(SD)	Post-operative Topical Glaucoma Medications(Mean)	Post-operative Topical Glaucoma Medications(SD)	Mean Reduction in Medications
Fea (2010) [28]	12	iStent + phaco	2	0.9	0.4	0.7	1.6
	24	Phaco only	1.9	0.7	1.3	1.0	0.6
Fernandez-Barrientos (2010) [39]	17	2 iStent + phaco	1.1	0.5	0	0	1.1
	16	Phaco only	1.2	0.7	0.7	1.0	0.5
Fea (2012) [40]	10	iStent + phaco	1.9	0.5	0.5	0.5	1.4
	14	Phaco only	2	0.5	1	0.5	1.0
Samuelson (2011) [26]	117	Phaco only	1.5	0.6	0.4	0.7	1.1
	123	iStent + Phaco	1.5	0.6	0.2	0.6	1.3

1. Based on literature [26,28,40], weighted mean reduction in topical glaucoma medications following Phaco only was 1.01.

2. Based on literature [26,28,40], weighted mean reduction in topical glaucoma medications following an iStent + Phaco was 1.33.

3. Based on literature [39], weighted mean reduction in topical glaucoma medications following 2 iStents + Phaco was 1.1.

**Table 5:** Reported number of pre- and post-operative topical glaucoma medications in studies considering phacoemulsification as a solo procedure.

Author (Year)	N	Baseline Topical Glaucoma Medications(Mean)	Baseline Topical Glaucoma Medications(SD)	Post-operative Topical Glaucoma Medications(Mean)	Post-operative Topical Glaucoma Medications(SD)	Mean Reduction in Medications
Altan-Yaycioglu (2009) [41]	26	1	0	1	0	0.0
Bowling (2009) [42]	51	1.5	0.5	0.6	0.5	0.9
Chang (2012) [43]	29	2.13	1.13	1.96	1.44	0.17
Chihara (2010) [44]	208	0.68	0.74	0.36	0.62	0.32
Guan (2013) [46]	103	1.5	1.3	1.5	1.3	0.0
Khondkaryan (2012) [48]	20	1.4	1.47	0.8	1.11	0.6
Klink (2005) [49]	36	0.8	0.5	0.8	0.5	0.0
Ismi (2013) [50]	21	1.48	0.5	1.24	0.5	0.24
Mathalone (2005) [52]	24	1.5	0.9	1.1	1.12	0.4
Park (2004) [54]	103	1.3	0.6	1	0.5	0.3
Poley (2010) [55]	124	1.3	0.7	1	0.7	0.3
Pohjalainene (2001) [56]	38	1.7	0.5	1.3	0.5	0.4
Sharma (2008) [58]	22	0.6	3.09	0.45	1.6	0.15
Shingleton (2006) [59]	55	1.1	0.5	1.1	0.7	0.0
Shingleton (2008a) [60]	137	0.12	0.03	0.09	0.24	0.03
Shingleton (2008b) [61]	240	1.6	0.9	1	1.1	0.6
Slabaugh (2014) [62]	157	1.85	1.01	1.92	1.07	-0.07

Weighted mean reduction in topical glaucoma medications following Phaco only was 0.23.

**Table 6:** Reported number of pre- and post-operative topical glaucoma medications in studies considering iStent with concurrent phacoemulsification.

Author (Year)	N	Intervention	Baseline Topical Glaucoma Medications(Mean)	Baseline Topical Glaucoma Medications(SD)	Post-operative Topical Glaucoma Medications(Mean)	Post-operative Topical Glaucoma Medications(SD)	Mean Reduction in Medications
Ahmed (2012) [63]	27	iStent + phaco	2.6	0.9	2.25	1.5	0.35
Arriola Villalobos (2013) [64]	20	2 iStents + Phaco	1.3	0.66	0.3	0.57	1.0
Belovay (2012) [65]	28	2 iStents + Phaco	2.8	0.8	1	0.6245	1.8
	25	3 iStents + Phaco	2.6	1.2	0.4	0.6245	2.2
Spiegel (2008) [66]	47	iStent + phaco	1.5	0.7	0.5	0.8	1.0
Patel (2013) [67]	40	iStent + phaco	2.3	1.7	0.59	1.7	1.71
Spiegel (2009) [68]	47	iStent + Phaco	1.5	0.7	0.5	0.8	0.96

1. Weighted mean reduction in topical glaucoma medications following an iStent + Phaco was1.05.

2. Weighted mean reduction in topical glaucoma medications following 2 iStents + Phaco was 1.46.

3. Weighted mean reduction in topical glaucoma medications following 3 iStents + Phaco was 2.2.

**Table 7:** Reported post-operative characteristics and complications of studies included in meta-analysis.

Author (Year)	Intervention	Patients Characteristics	Complications reported (rate %)	Race distribution (%)
Ahmed (2012) [63]	iStent + phaco	-	Stent occlusion (11.1), hyphema (3.7), IOP raise above 10 mmHg (48.1)	-
Arriola Villalobos (2013) [64]	2 iStents + phaco	PoM (75)	transient raise in IOP above 30 mmHg (15)	Caucasian (100)
Belovay (2012) [65]	2 iStents + Phaco	PEV (64), PoM (46)	Blockage of the opening of the stent lumen in 8 eyes (15), hyphema (2), stent malposition (2)	Caucasian (69), African American (15), South Asian (8), Far East Asian (8)
	3 iStents + Phaco	PEV (76), PoM (72)	-	
Carrillo, M(2005) [69]	Phaco only	-	Poor visual outcomes (6), CME (3)	-
Fea (2010) [28]	iStent + Phaco	-	Stent malposition (6)	Caucasian (100)
	Phaco only	PoM (67)	Capsule rupture (4.2)	-
Fernandez-Barrientos (2010) [39]	2 iStents + phaco	-	Stent malposition (18)	-
Fea, A(2012) [40]	iStent + Phaco	-	No secondary surgical interventions were reported	Caucasian (100)
	Phaco only	-	and no other adverse events or untoward findings were reported in either group.	
Guan (2013) [46]	Phaco only	-	-	Caucasian (73), African American (20), Other (7)
Spiegel (2008) [66]	iStent + Phaco	PoM (70)	Anterior chamber collapse (2), vitreous wick incarcerated in paracentesis (2), stent malposition (2.13), stent occlusion (14.89)	Caucasian (97.9), Hispanic (2.1)
Klink (2005) [49]	Phaco only	-	None	-
Mathalone (2005) [52]	Phaco only	-	-	African American (14.3)
Patel (2013) [67]	iStent + Phaco	PoM (66)	Hyphema (2.3)	-
Samuelson (2011) [26]	Phaco alone	PEV (44), PoM (35)	PCO (7), subconjunctival hemorrhage (2), epiretinal membrane (1)iritis (5), dry eye (2), allergic conjunctivitis (2), mild pain (2)	Caucasian (71), Hispanic (13), African American (14)
	iStent + Phaco	PEV (45), PoM (15)	Stent malposition (3), stent occlusion (4), CME (1), PCO (3), subconjunctival hemorrhage (2), epiretinal membrane (2), iris atrophy (2), iritis (1), dry eye (1)	
Sharma (2008) [58]	Phaco only	-	-	Caucasian (53.85), Asian (19.23), African American (7.7)
Shingleton (2008a) [61]	Phaco only	-	Retinal detachment (7.3), CME (7.3), IOL decentralization (7.3)	-
Slabaugh (2014) [62]	Phaco only	-	-	Caucasian (75.2), Asian (12.1), African American (16)
Spiegel (2009) [68]	iStent + Phaco	PoM (50)	Stent malposition (17), stent occlusion (14)	-

PoM = % of patients off medications

PEV = % of eyes 20/40 and better vision

**Table 8:** Reported pre- and post-operative best corrected visual acuity in studies included for meta-analysis.

Author (Year)	Intervention	Mean Pre-operative BCVA (LogMAR)	SD of Pre-operative BCVA (LogMAR)	Mean Post-operative BCVA (LogMAR)	SD of Post-operative BCVA (LogMAR)
Arriola Villalobos (2013) [64]	2 iStent + phaco	0.4	0.92	0.096	0.77
Carrillo, M (2005) [69]	Phaco only	0.62	-	1.15	-
Chihara (2010) [44]	Phaco only	0.3	0.54	0.041	0.41
Damji (2006) [45]	Phaco only	0.52	0.31	-	-
Fea (2012) [40]	iStent + Phaco	0.36	-	0.11	-
	Phaco only	0.44	-	0.11	-
Klink (2005) [49]	Phaco only	0.85	-	-	-
Ismi (2013) [50]	Phaco only	0.92	0.93	0.33	0.499
Merkur (2001) [53]	Phaco only	0.53	0.27	-	-
Patel (2013) [67]	iStent + Phaco	0.53	-	0.23	-
Park (2004) [54]	Phaco only	0.36	0.44	0.092	0.74
Samuelson (2011) [26]	Phaco only	0.36	0.23	-	-
	iStent + Phaco	0.36	0.23	-	-
Shingleton (2006) [59]	Phaco only	0.59	0.41	0.31	0.3
Shingleton (2008a) [61]	Phaco only	0.24	0.35	0.62	0.62
Shingleton (2008b) [60]	Phaco only	0.54	0.02	0.18	0.03
	Phaco only	0.48	0.02	0.18	0.03
Slabaugh (2014) [62]	Phaco only	0.394	0.29	0.098	0.2

### Publication Bias

**Fig 2:**
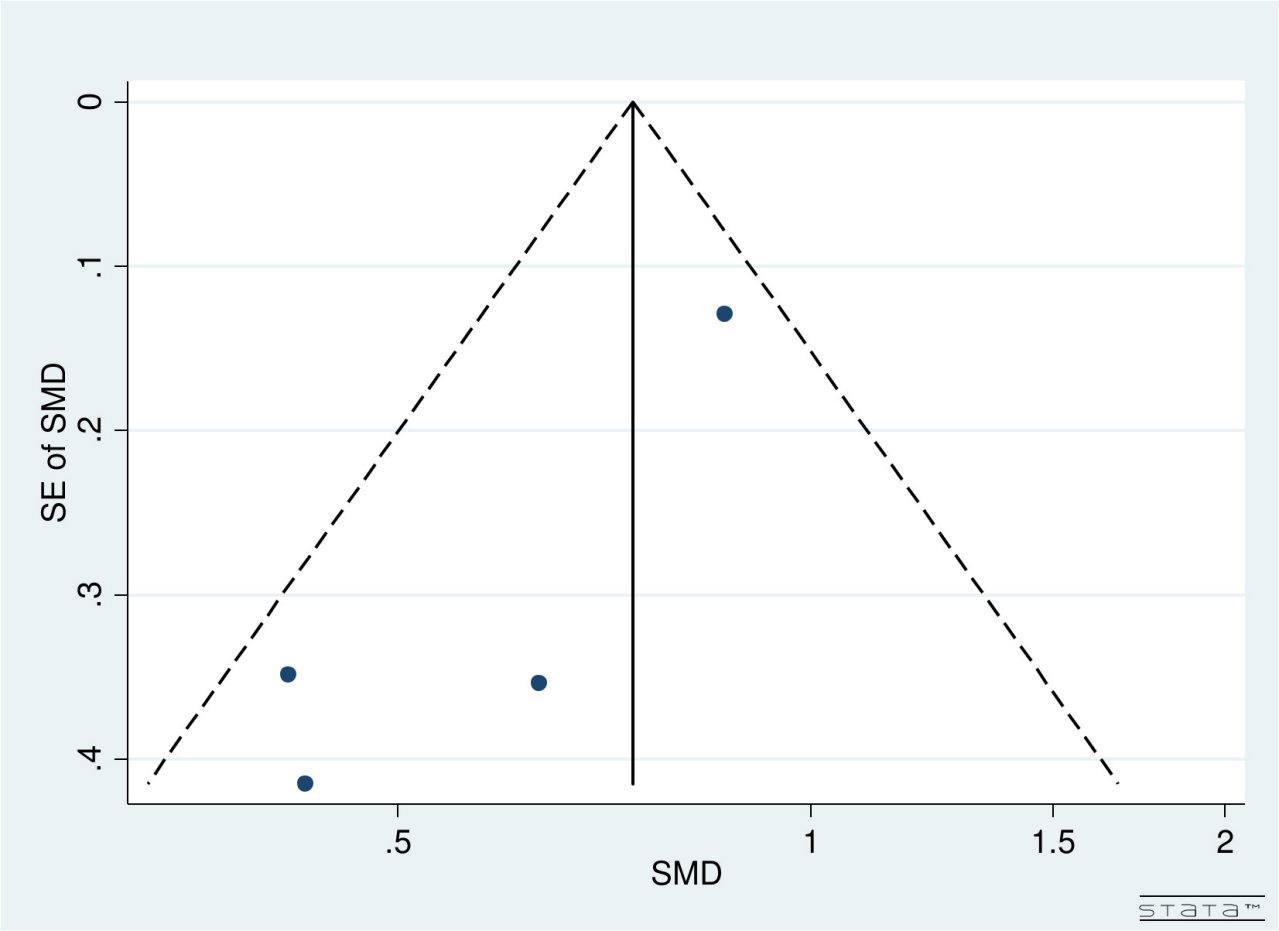
Funnel plot for pre- and post-operative IOPR% for studies comparing iStent insertion with phacoemulsification versus phacoemulsification as a solo procedure.

**Fig 3:**
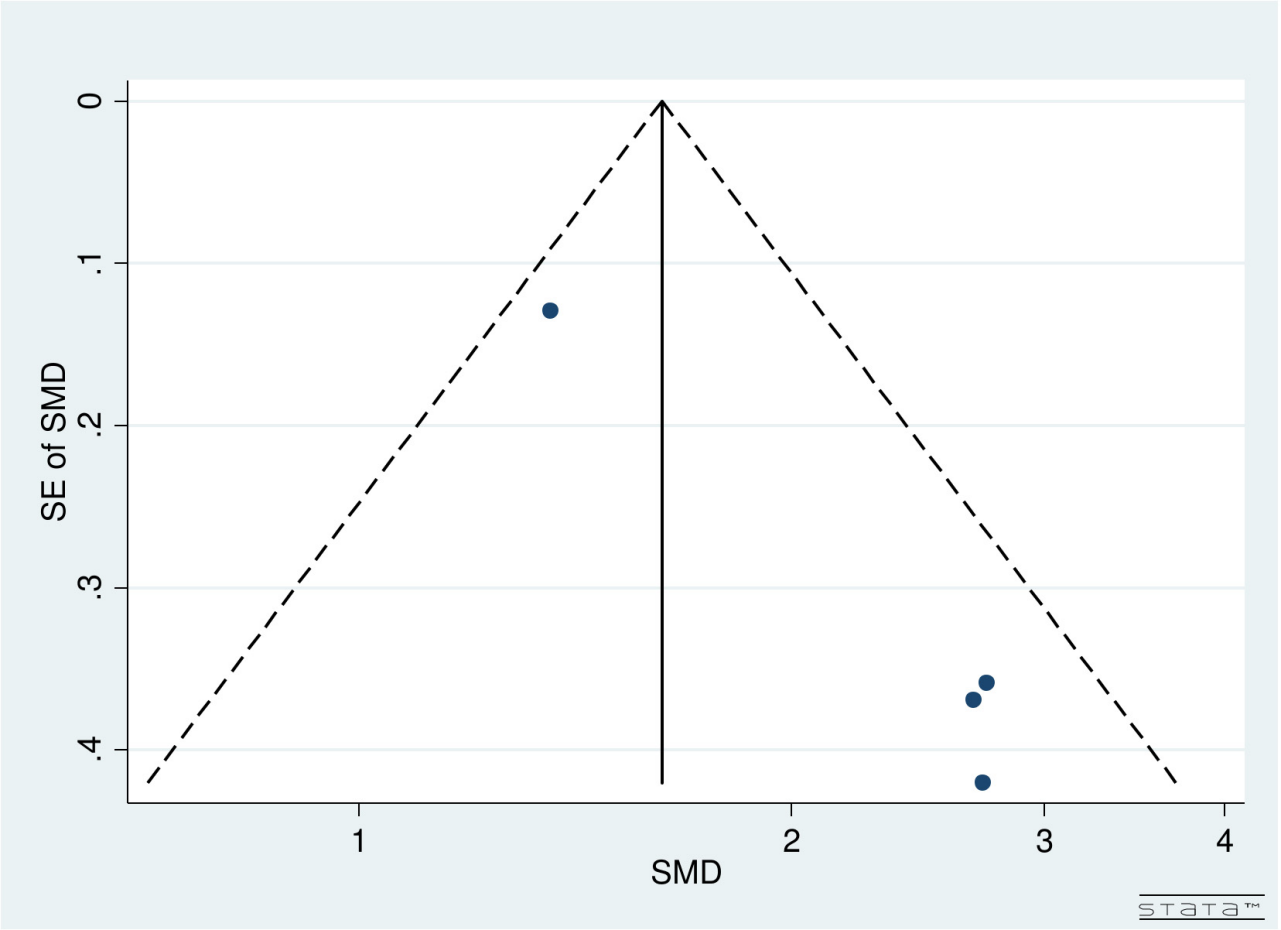
Funnel plot for pre- and post-operative number of topical glaucoma medications for studies comparing iStent insertion with phacoemulsification versus phacoemulsification as a solo procedure.

Visual inspection of funnel plots by follow-up and number of iStents implanted for both pre- and post-operative IOPR% and topical medications did not reveal any asymmetry (Figs 2 and 3).

### Effect on IOPR%

A 4% IOP reduction (IOPR%) from baseline occurred following phacoemulsification as a solo procedure compared to 9% following an iStent implant with phacoemulsification, and 27% following 2 iStents implants with phacoemulsification (Table 1) based on 4 studies comparing phacoemulsification only and iStent with concurrent phacoemulsification. Based on 22 studies considering phacoemulsification as a solo procedure in patients with glaucoma and cataract, a 31% IOP reduction from baseline occurred (Table 2). This reflects significant between study variations due to numerous factors such as patient’s income status, socio-economic status, previous ocular and non-ocular surgeries, family history, other ocular and non-ocular diseases, pre-operative and post-operative medications, number of medications, comorbidities, etc. influencing the estimates in the original studies. A 26% IOP reduction from baseline occurred following an iStent implant with phacoemulsification, 18% following 2 iStents implants with phacoemulsification, and 20% following 3 iStents implants with phacoemulsification based on 6 studies considering iStent implant with concurrent phacoemulsification (Table 3).

**Fig 4:**
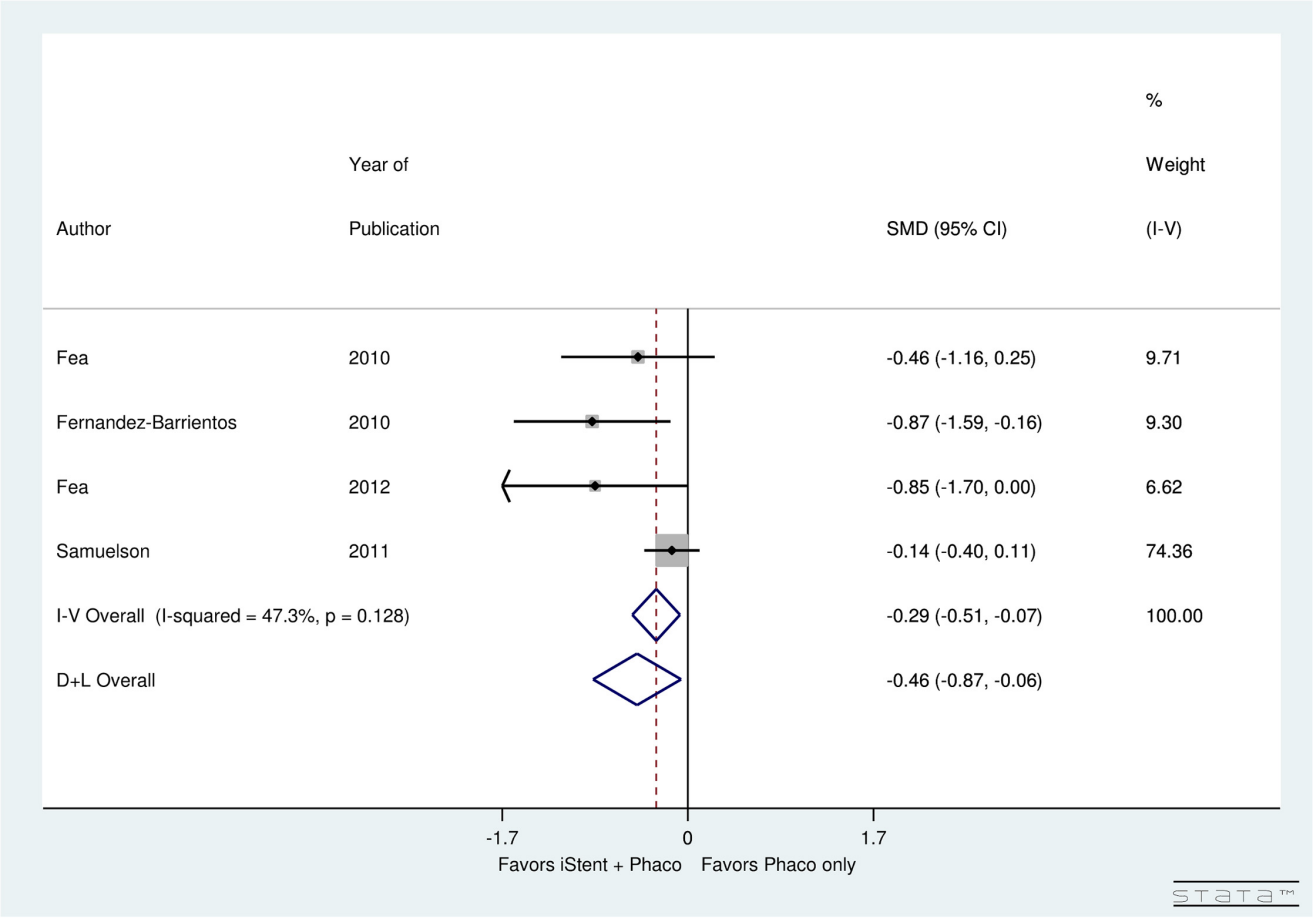
Forest plot for difference in post-operative IOPR% for studies comparing iStent insertion with phacoemulsification versus phacoemulsification as a solo procedure.

Four studies examined the post-operative IOPR% for phacoemulsification with iStent compared to phacoemulsification alone for patients with glaucoma and cataract. Non-significant (*p* = 0.128) heterogeneity was found between these studies (I^2^ = 47.3%). A significant decrease in the post-operative IOPR% was found in the phacoemulsification with iStent group compared to phacoemulsification only group (SMD = -0.46, 95% CI: [-0.87, -0.06]) (Fig 4).

### Effect on Topical Glaucoma Medication Use

A weighted mean reduction in the number of glaucoma medications per patient was 1.01 following phacoemulsification alone compared to 1.33 after one iStent implant with phacoemulsification, and 1.1 after 2 iStent implants with phacoemulsification based on 4 studies comparing phacoemulsification only and iStent with concurrent cataract procedure (Table 4). Based on 22 studies considering phacoemulsification as a solo procedure, a weighted mean reduction in the number of glaucoma medications per patient was 0.23 (Table 5). Based on 6 studies considering iStent with concurrent phacoemulsification, a weighted mean reduction in the number of glaucoma medications per patient was 1.05 after an iStent implant with phacoemulsification, 1.46 after 2 iStents implants with phacoemulsification, and 2.2 after 3 iStents implants with phacoemulsification (Table 6).

**Fig 5:**
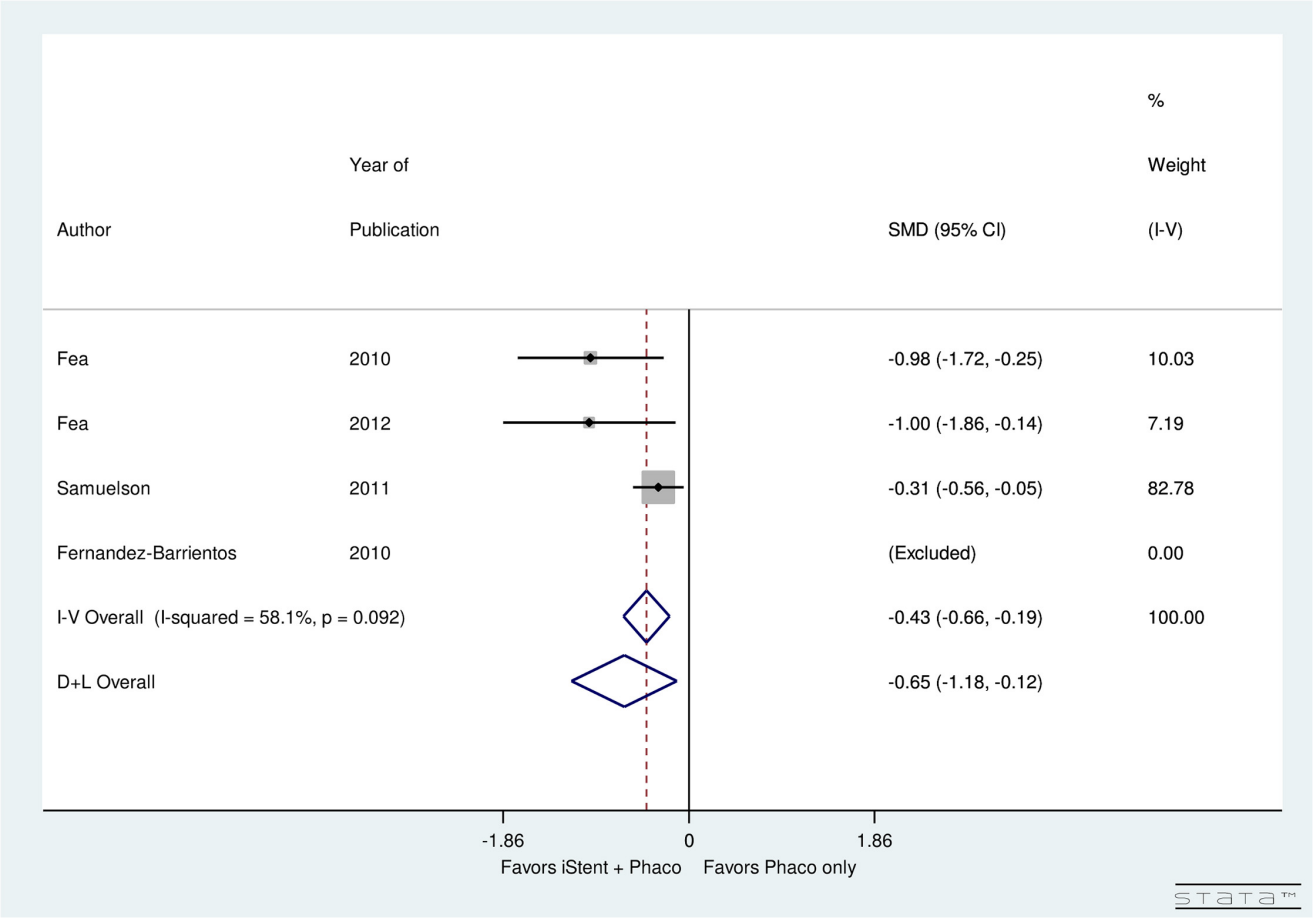
Forest plot for difference in post-operative number of topical glaucoma medications of iStent with phacoemulsification versus phacoemulsification only.

Four heterogeneous studies (I^2^ = 58.1%) performed a direct comparison of the number of post-operative topical glaucoma medications used after phacoemulsification with iStent insertion versus phacoemulsification alone. When compared to the phacoemulsification alone group, there was a significant decrease in the number of medications used post-operatively for the phacoemulsification with iStent group (SMD = -0.65, 95% CI: [-1.18, -0.12]) (Fig 5).

**Fig 6:**
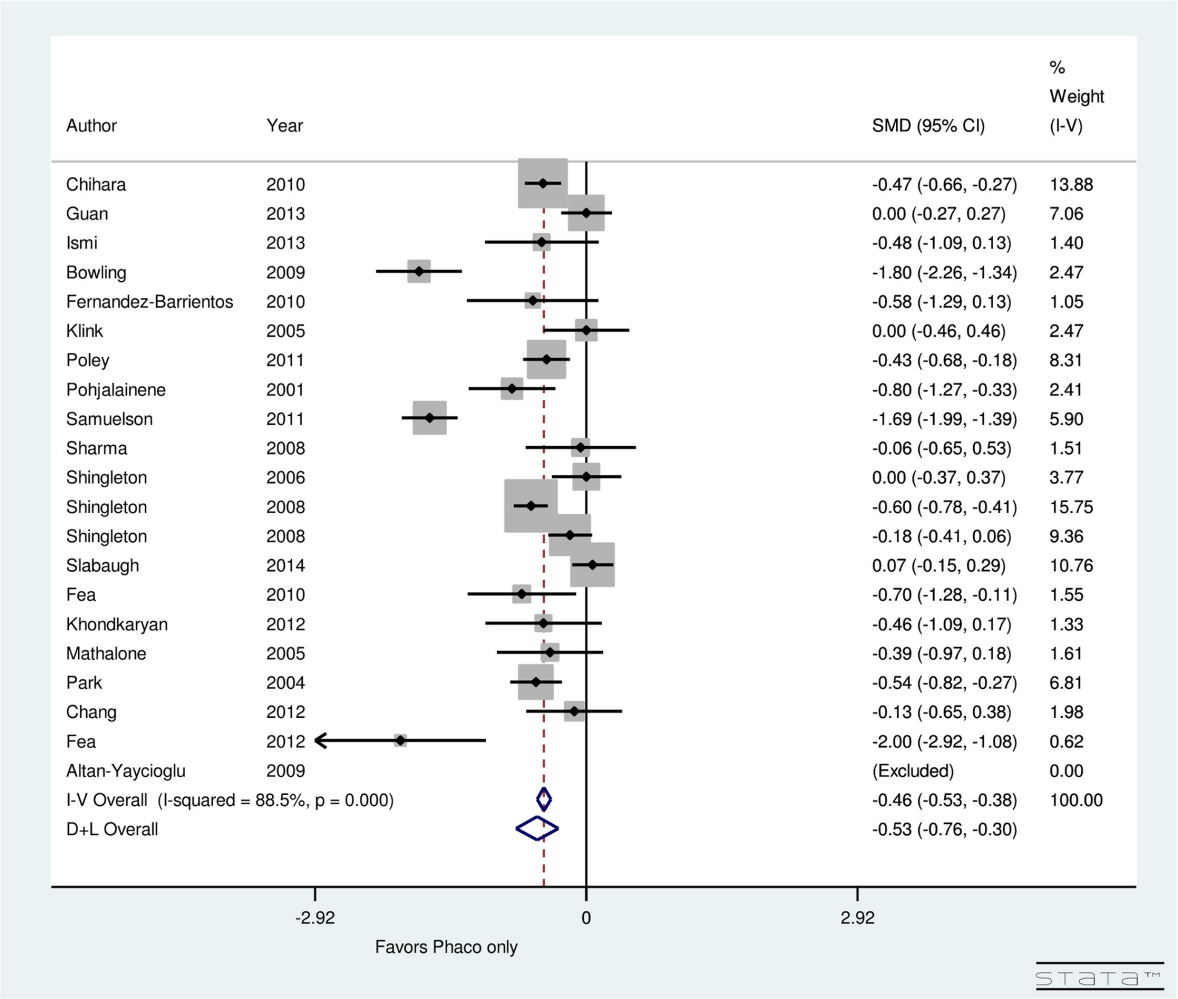
Forest plot for pre- and post-operative number of topical glaucoma medications for studies examining phacoemulsification as a solo procedure.

**Fig 7:**
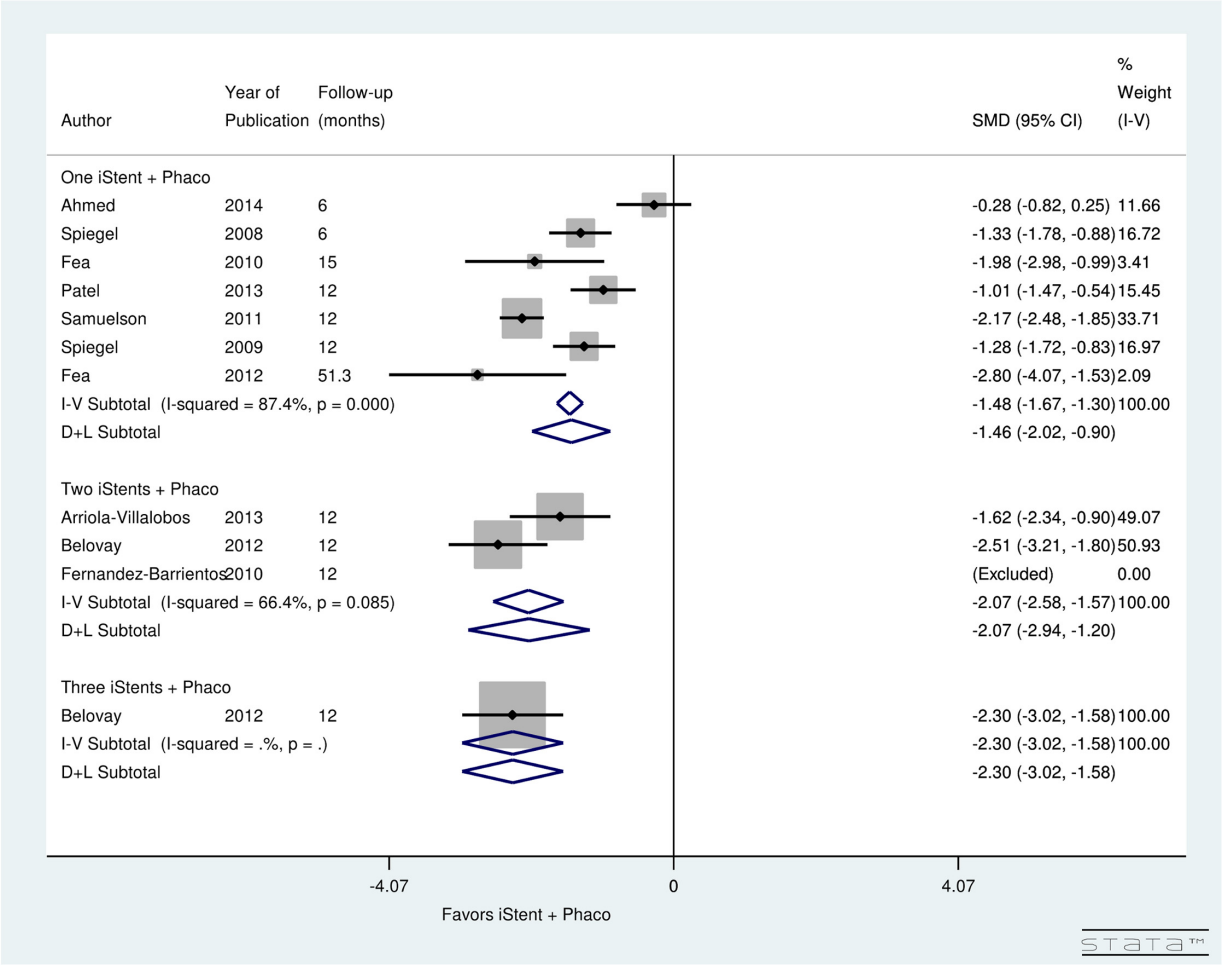
Forest plot for pre- and post-operative number of topical glaucoma medications for studies examining iStent insertion with simultaneous phacoemulsification.

Significant (*p* = 0.0) heterogeneity was observed between studies evaluating pre- and post-operative topical glaucoma medication use for patients undergoing phacoemulsification only (I^2^ = 88.5%). Results showed a significant decrease in the postoperative number of medications due to phacoemulsification only (SMD = -0.53, 95% CI: [-0.76 to -0.30]) (Fig 6). Due to significant (*p* = 0.0) heterogeneity (I^2^ = 87.4%), a random-effect computation was used to analyze the studies examining medication use after simultaneous iStent insertion and phacoemulsification. The analysis showed a significant decrease in the number of post-operative medications after iStent with phacoemulsification (SMD = -1.46, 95% CI: [-2.02 to -0.90]). The three studies examining the insertion of two iStent devices at the time of phacoemulsification displayed non-significant (*p* = 0.085) heterogeneity (I^2^ = 66.4%). The pooled data suggested a significant difference between the pre- and post-operative number of medications after the insertion of two iStents at the time of phacoemulsification (SMD = -2.07, 95% CI: [-2.94 to -1.20]). A single study examined the insertion of three iStent devices with concurrent phacoemulsification; the investigators found a significant decrease in the use of medications post-operatively (SMD = -2.30, 95% CI: [-3.02 to -1.58]) (Fig 7).

**Fig 8:**
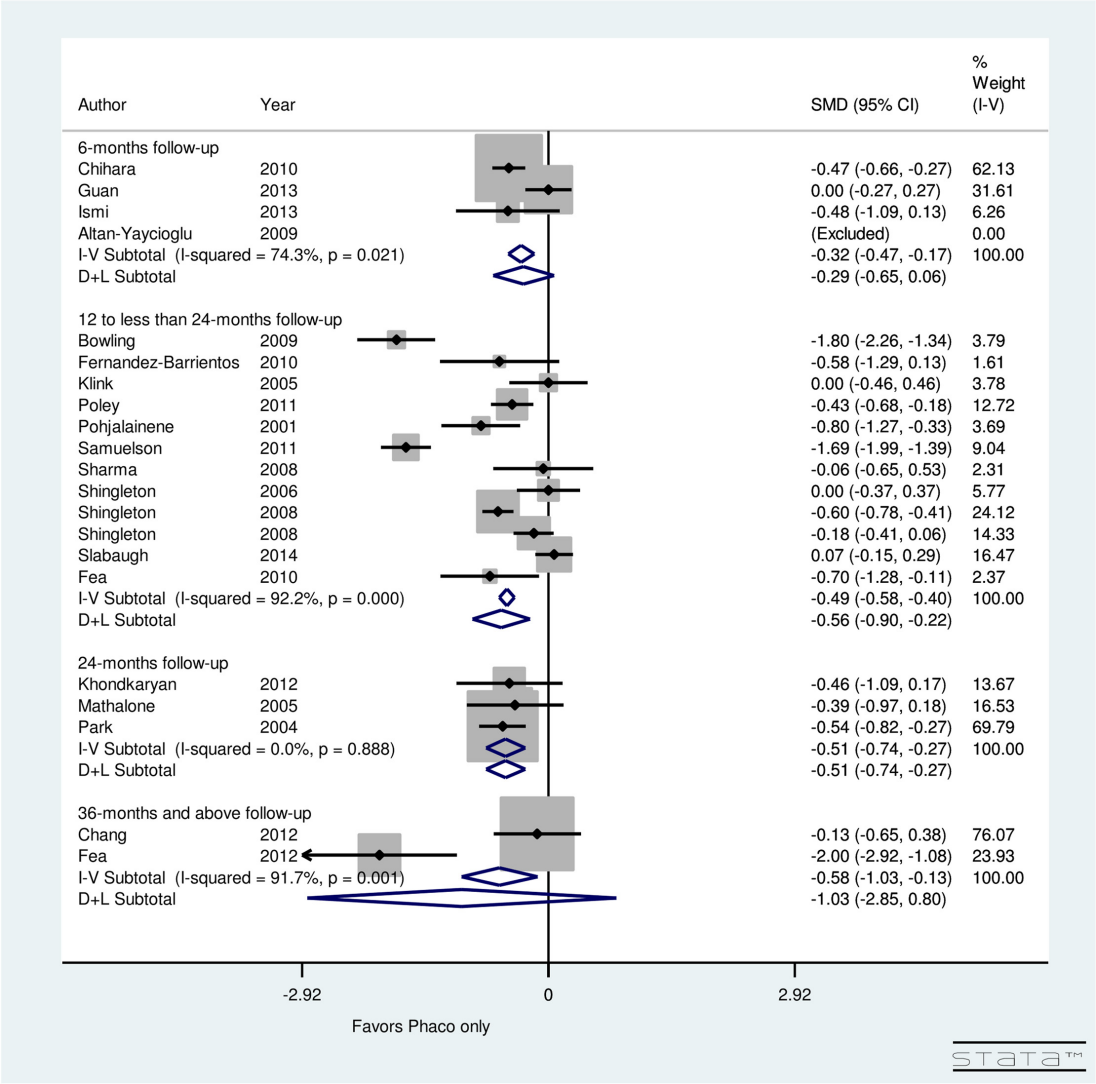
Forest plot for pre- and post-operative number of topical glaucoma medications by follow-up (months) for phacoemulsification only group.

Fig 8 presents the post-operative change in topical glaucoma medication use, stratified by follow-up period, for patients undergoing phacoemulsification only. There was non-significant (*p* = 0.021) heterogeneity between 4 studies examining 6-months follow-up (I^2^ = 74.3%), 12 studies with a follow-up of 12 to less than 24 months (I^2^ = 92.2%), and 3 studies with a 24-months follow-up (I^2^ = 0.0%). The meta-analysis suggested that the maximum reduction in number of medications occurred during 12 to less than 24 month time period (SMD = -0.56, CI: [-0.9, -0.22]). Additionally, non-significant reduction in number of medications occurred at 6 months (SMD = -0.29, CI: [-0.65, 0.06]) and at 36 months (SMD = -1.03, CI: [-2.85, 0.8]). However, significant reduction occurred in topical glaucoma medications at 24 months (SMD = -0.51, CI: [-0.74, -0.27]).

**Fig 9:**
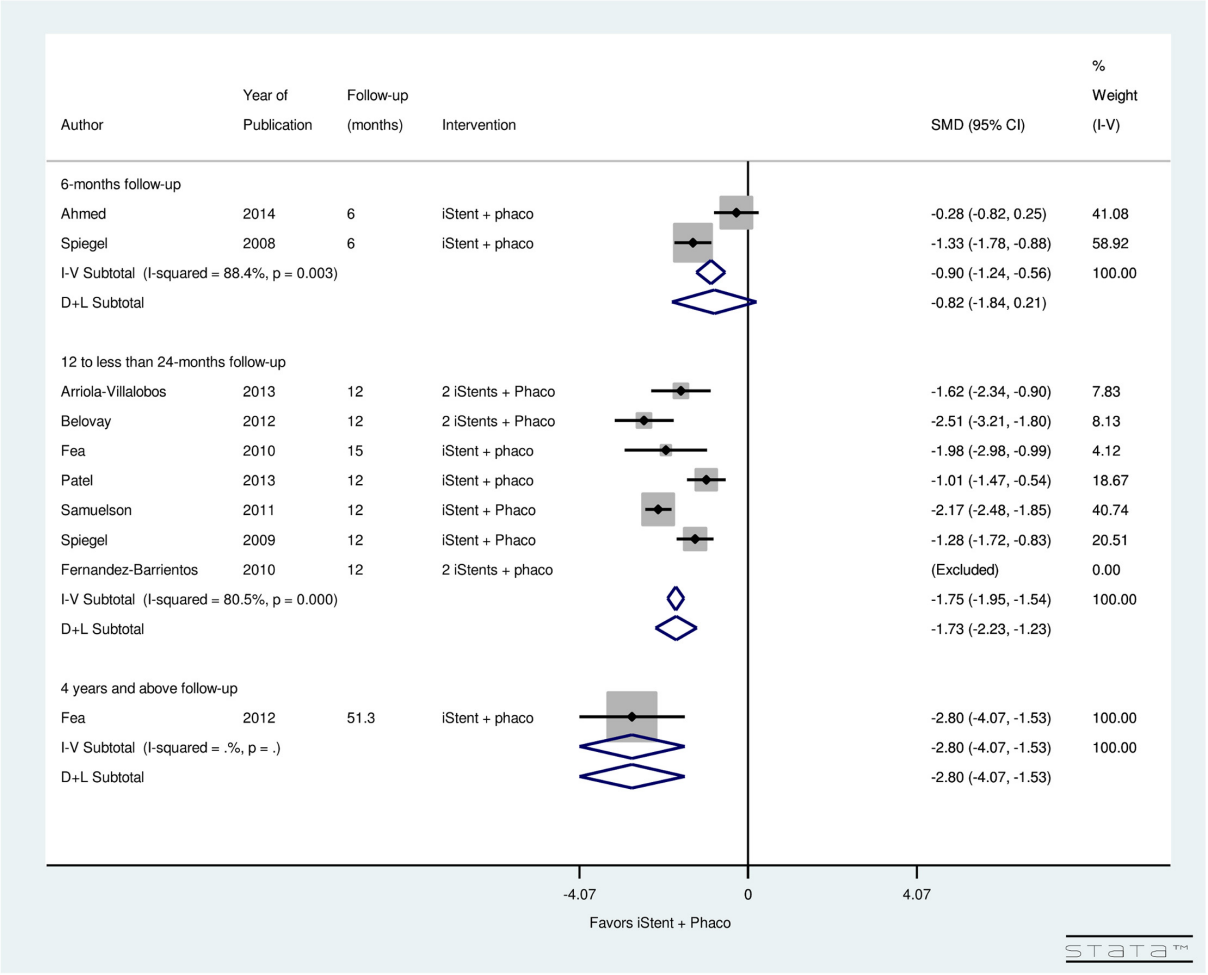
Forest plot for pre- and post-operative number of topical glaucoma medications by follow-up (months) for iStent and simultaneous phacoemulsification group.

Fig 9 presents the post-operative change in topical glaucoma medication use, stratified by follow-up period, for patients undergoing phacoemulsification with simultaneous iStent insertion. Significant heterogeneity was found between 2 studies with a follow-up of 6-months (I^2^ = 88.4%), 7 studies with a follow-up of 12 to less than 24 months (I^2^ = 80.5%), and 1 study with a follow-up of 4 years and above (I^2^ = 0.0%). Results showed non-significant decrease in post-operative number of medications in 6-months (SMD = -0.82, CI: [-1.84, 0.21]). Significant decrease in post-operative number of medications was found from 12 to 24 months (SMD = -1.73, CI: [-2.23, -1.23]) and after 4 years of iStent with phacoemulsification surgery (SMD = -2.8, CI: [-4.07, -1.53]).

## Discussion

The aggregated results presented in this meta-analysis indicate that both iStent implantation with concurrent phacoemulsification and phacoemulsification alone result in a significant decrease in IOP and topical glaucoma medications. In terms of both reductions, iStent with phacoemulsification significantly outperforms phacoemulsification alone.

A 4% IOP reduction (IOPR%) from baseline occurred following phacoemulsification as a solo procedure compared to 9% following an iStent implant with phacoemulsification, and 27% following 2 iStents implants with phacoemulsification. A weighted mean reduction in the number of glaucoma medications per patient was 1.01 following phacoemulsification alone compared to 1.33 after one iStent implant with phacoemulsification, and 1.1 after 2 iStent implants with phacoemulsification.

The maximum reduction in the number of medications after phacoemulsification alone occurred at 12 months up to until 24 months. A significant reduction in the number of medications following phacoemulsification alone occurred at 24 months. Combined iStent with phacoemulsification significantly reduced the number of post-operative medications by 12 months of surgery which remained significantly reduced until 4 years of follow-up.

There was substantial heterogeneity among studies and therefore, a random-effect model was developed when required. A number of factors likely contributed to the heterogeneity including inconsistency in the differences in the study population, technical aspects of iStent implantation, technical differences in phacoemulsification, differential peri-operative management, differences in the study population, potential variability in facilities to perform surgeries, variable follow-up periods, rates of complications, year the surgeries were performed, attention to identification of collector channels as well as potential biases related to industry funding of studies. Although funding source is a potential bias it is recognized that peer-reviewed funding for device trials is exceedingly difficult to obtain. Recognizing and appreciating this bias is sufficient.

Limitations are inherent in any systematic review and meta-analysis due to the variability in the availability of published data. First, only limited information was available on pre-operative and post-operative contrast sensitivity, spherical equivalent, refractive status, stereopsis, and astigmatism and thus these parameters were excluded from the quantitative analysis. Second, meta-analysis of observational studies is influenced by inherent biases in the included articles. [38] For example, there could be other factors such as income status, socio-economic status, previous ocular and non-ocular surgeries, family history, other ocular and non-ocular diseases, pre-operative and post-operative medications, number of medications, comorbidities such as high blood pressure, diabetes, stroke, heart conditions, etc. influencing the estimates in the original studies. Third, some studies were excluded due to lack of necessary information. Data from these studies could have influenced the results.

As devices can be approved and brought to market with relatively few, and often smaller studies than medications, systematic reviews and meta-analyses provide the ability to analyze available data collectively with some degree of neutralization of the biases that may temper smaller studies. In chronic and prevalent diseases like glaucoma, for which the cause and cure remain unknown, innovations may represent a major step forward in management. However, without mechanisms to provide access and implementation, the potential benefits in patient care continue to remain unknown. Unfortunately criteria for approval at multiple levels, which are often influenced significantly by cost, represent potential barriers to access. Systematic reviews and meta-analyses may help provide an evidence-based foundation that can be used by health care systems and administrators to guide important decisions regarding access to innovation in a cost-driven era of health care management.

## Supporting Information

S1 File PRISMA 2009 Checklist. (DOC)

S2 File Search Strategy for EMBASE. (DOCX)

S3 File Level 1, 2, and 3 screening questions. (DOCX)
