# Supplementary material for: iStent with Phacoemulsification versus Phacoemulsification Alone for Patients with Glaucoma and Cataract: A Meta-Analysis
Source: PLoS One. 2015 Jul 6;10(7):e0131770. doi: 10.1371/journal.pone.0131770 (PMC4492499; doi:10.1371/journal.pone.0131770)
Supplement: S2 File — (DOCX) [file pone.0131770.s002.docx]

**S2: SEARCH STRATEGY for EMBASE**

Database: Embase Classic+Embase <1947 to 2014 March 21>

Search Strategy:

--------------------------------------------------------------------------------

1 Intraocular hypertension/ or Open angle glaucoma/ (18734)

2 (Ocular hypertens* or Intraocular hypertens* or Intra-ocular hypertens* or Suspect glaucoma* or hydrophthalmos or Pigmentary Glaucoma* or Simple Glaucoma* or Open-Angle Glaucoma* or Glaucoma simplex or Open Angle Glaucoma* or Wide angle glaucoma*).mp. (22178)

3 exp animals/ or invitro/ or (animal or rat$ or mouse or mice or "in vitro").mp. (21012297)

4 human/ or men/ or women/ or male/ or female/ or (man or men or woman or women or male$ or female$ or human).mp. (17246228)

5 Adolescent/ or exp Child/ or exp Infant/ or adolescen$.mp. or child$.mp. or infant.mp. or teenage$.mp. (3279659)

6 exp Adult/ or adult$.mp. or aged.mp. or elder$.mp. or middle age.mp. (6103808)

7 6 not 5 (4347933)

8 4 not 5 (13966569)

9 1 and 4 (21178)

10 limit 18 to (english language and yr="2000 -Current") (20)
