# Supplementary material for: iStent with Phacoemulsification versus Phacoemulsification Alone for Patients with Glaucoma and Cataract: A Meta-Analysis
Source: PLoS One. 2015 Jul 6;10(7):e0131770. doi: 10.1371/journal.pone.0131770 (PMC4492499; doi:10.1371/journal.pone.0131770)
Supplement: S3 File — (DOCX) [file pone.0131770.s003.docx]

**S3: LEVEL 1, 2, AND 3 SCREENING QUESTIONS**

**Level 1 screening**

1. Does the study look at selective laser trabeculoplasty (SLT) or laser trabeculoplasty in adults with glaucoma?
   1. Yes
   2. No
   3. Unclear
2. Is this a research study (not an editorial, opinion, case report or a review article)?
   1. Yes
   2. No
   3. Unclear

**Level 2 Screening**

1. Does the study look at efficacy/best corrected visual acuity/IOP levels/any other outcomes or side-effects/complications or cost of Phacoemulsification with iStent or phaco alone in adults with OAG?
   1. Yes
   2. No
   3. Unclear
2. Is there an adequate follow-up to assess efficacy or side-effects or cost of phacoemulsification with iStent or phaco alone?
   1. Yes
   2. No
   3. Unclear
3. Does the study consider the sample size of 20 or more patients?
   1. Yes
   2. No
   3. Unclear
4. Is this a research study (not a pilot study or a survey considering percentage of people suffering from glaucoma)?
   1. Yes
   2. No
   3. Unclear
5. Have patients in this study not been on ALT or trabeculectomy?
   1. Yes
   2. No
   3. Unclear

**Level 3 Screening**

1. Are complications explicitly discussed and included in the paper?
2. Yes
3. No
4. Unclear
5. Are complications and outcomes (ie IOP, vision, compliance, quality of life) or cost or probability of occurrence of an outcome/complication explicitly discussed and included in the paper?
6. Yes
7. No
8. Unclear
